# Hypertension after preeclampsia and relation to the C1114G polymorphism (rs4606) in *RGS2*: data from the Norwegian HUNT2 study

**DOI:** 10.1186/1471-2350-15-28

**Published:** 2014-03-05

**Authors:** Anne Stine Kvehaugen, Øyvind Melien, Oddgeir L Holmen, Hannele Laivuori, Ralf Dechend, Anne Cathrine Staff

**Affiliations:** 1 From the Department of Obstetrics and Department of Gynecology, Oslo University Hospital, Ulleval, Oslo, Norway and Faculty of Medicine, University of Oslo, Oslo, Norway; 2 The Norwegian Directorate of Health, Oslo, Norway; 3 HUNT Research Centre, Department of Public Health and General Practice, Norwegian University of Science and Technology, Levanger, Norway; 4 Haartman Institute, Medical Genetics, University of Helsinki Helsinki, Finland and Department of Obstetrics and Gynecology, Helsinki University Central Hospital, Helsinki, Finland; 5 Experimental and Clinical Research Center, a joint cooperation between the Charite Medical Faculty and the Max-Delbrueck Center for Molecular Medicine and Helios Clinic Berlin-Buch, Berlin, Germany

**Keywords:** Preeclampsia, Hypertension, Polymorphism, G proteins, RGS2, Angiotensin II

## Abstract

**Background:**

Preeclampsia is associated with an increased risk of hypertension later in life. The regulator of G protein signaling 2 negatively regulates several vasoconstrictors. We recently demonstrated an association between preeclampsia and the CG or GG genotype of the C1114G polymorphism (rs4606) of the regulator of G protein signaling 2 gene. Here, we examined the polymorphism with respect to the development of hypertension after pregnancy.

**Methods:**

We genotyped 934 women on average 15.1 years after preeclampsia and 2011 age matched women with previous normotensive pregnancy. All women in this study were retrospectively recruited from the Nord-Trøndelag Health Study (HUNT2). Information from HUNT2 was linked to the Medical Birth Registry of Norway to identify women with a history of preeclampsia and women without a history of preeclampsia.

**Results:**

No significant association was found between hypertension (blood pressure ≥140/90 mmHg and/or taking antihypertensive drugs) and the polymorphism in crude analysis (OR (95% CI): CG genotype: 1.07 (0.90-1.27); GG genotype: 1.23 (0.90-1.67)). However, in a minimally adjusted model (age and BMI adjusted), a significant association between the GG genotype and hypertension was found (OR (95% CI): 1.49 (1.05-2.11)). This association remained significant also after adjustment for a history of preeclampsia (OR (95% CI): 1.46 (1.02-2.09)), but not in a model adjusted for multiple other variables (OR (95% CI): 1.26 (0.82-1.94)). In multivariate, but not in crude, analysis, the GG genotype of rs4606 (OR (95% CI): 1.93 (1.05-3.53)) was significantly and independently associated with severe hypertension later in life, defined as systolic blood pressure ≥160 mmHg (stage 2 hypertension) and/or taking antihypertensive drugs. A significant association was also found for the merged CG and GG genotypes (OR (95% CI): 1.43 (1.02-2.00)). Moreover, an interaction with physical activity was found. A history of preeclampsia was a significant and independent predictor of either definition of hypertension, both in crude and adjusted analyses.

**Conclusion:**

Women carrying the rs4606 CG or GG genotype are at elevated risk for developing hypertension after delivery. Physical activity may interact with the association. Preeclampsia remains an independent risk factor for subsequent hypertension after adjusting for this polymorphism and classical CVD risk factors.

## Background

Preeclampsia affects approximately 2-8% of all pregnancies [1] and is clinically manifested as proteinuria and hypertension after 20 weeks of gestation [2]. Preeclampsia is recognized as a risk factor for hypertension and cardiovascular disease (CVD) later in life [3], with early-onset (with delivery prior to gestational week 34) and recurrent episodes of preeclampsia being even more strongly associated with later CVD [4]. The mechanistic links between preeclampsia and future health are incompletely elucidated. Common predisposition, genetic or environmental, that could contribute to the development of both preeclampsia and hypertension or CVD later in life, is a possible explanatory link. In line with this, classical CVD risk factors such as obesity, dyslipidemia, insulin resistance, family history and endothelial dysfunction are also associated with preeclampsia [3]. A pregnancy complicated by preeclampsia as a direct causal factor, or an “amplifying factor” for the association, can also not be ruled out.

The regulator of G protein signaling 2 (RGS2) acts as a GTP-ase activating protein for Gαq proteins and is thereby involved in controlling the termination of vasoconstrictor signaling initiated by ligands such as Angiotensin II (Ang II) [5]. Hercule et al. showed that *RGS2* deletion promotes Ang II-dependent hypertension primarily through an increase of myogenic tone and vasoreactivity, probably by sensitization of Ang II type 1 receptor (AT1-receptor) [6]. The G allele of a polymorphism (rs4606) in the three prime untranslated region of *RGS2* has previously been associated with reduced *RGS2* expression [7]. This study, which included both men and women, also found that those with hypertension more often carried the G allele compared to normotensive controls [7]. We recently identified the CG or GG genotype to be more common among women with preeclampsia compared to women without preeclampsia [8]. This difference in the genotype distribution between cases and controls could possibly also contribute to the increased risk of hypertension later in life in women with a history of preeclampsia. Based on the majority of our previous study population, the population-based HUNT2 study, we wanted to explore whether rs4606 in *RGS2* is associated with development of hypertension after pregnancy, which to our knowledge has not been investigated previously.

## Methods

### Study population

All women in this study (n = 2945) were retrospectively recruited from the phase 2 of the Nord-Trøndelag Health Study (the HUNT2 Survey). HUNT2 is a large multipurpose health survey conducted in 1995–1997, where all residents ≥ 20 years old living in Nord-Trøndelag county, Norway, were invited, and a total of 75.5% (n = 35,280) of the invited women participated [9]. Clinical information was collected from self-reported questionnaires, which included questions on the use of antihypertensive medication, physical activity, smoking and diseases such as CVD and diabetes mellitus. A standardized clinical examination included height, weight and blood pressure measurements. Blood pressure was measured using an automatic oscillometric method (Dina map 845XT; Critikon) after participants had rested in the sitting position for a minimum of two minutes. Three subsequent readings were taken at intervals of one minute [9] and the average of the second and third readings were used in the present study. Serum lipids (including total cholesterol and high density lipoprotein (HDL)) were measured in non-fasting venous blood samples and DNA was isolated from peripheral blood [9].

We linked information from HUNT2 with the Medical Birth Registry of Norway (MBRN). MBRN registers all deliveries in Norway since 1967 by compulsory notification, and the diagnosis of preeclampsia in the MBRN is based on the individual delivery reports from the delivering units. Delivery units in Norway presently use the American College of Obstetricians and Gynecologist (ACOG) criteria for a diagnosis of preeclampsia; systolic blood pressure ≥140 mmHg or diastolic blood pressure ≥90 mmHg that occurs after 20 weeks of gestation in a previously normotensive woman, combined with proteinuria (urinary excretion of ≥ 0.3 g protein in a 24-hour urine specimen) [2]. As cases, we selected individuals with a diagnosis of preeclampsia in MBRN prior to participation in HUNT2, who also had an available DNA sample at HUNT Biobank. A validation study of the diagnosis of preeclampsia against the medical records for participants in the HUNT2 study that were previously selected for two different genetic studies of preeclampsia has recently been published [10]. In this study, a diagnosis of preeclampsia was confirmed in 88.3% of pregnancies during 1967–2002, and in 63.6% of pregnancies during 1967–2005, using the broader and traditional criteria of one measurement of hypertension and proteinuria versus today’s criteria of two repeated measurements of proteinuria and hypertension respectively [10], suggesting an acceptable validation of the preeclampsia diagnosis in the HUNT2 study. Absence of a medical chart documented proteinuria accounted for more than 70% of a non-confirmed preeclampsia diagnose [10], not necessarily excluding a history of proteinuria and thereby a diagnosis of preeclampsia.

As controls (≈2:1 controls per case), we selected women with at least one delivery prior to participating in the HUNT2 study, who also had available DNA at HUNT Biobank and never were registered with preeclampsia in the MBRN. Hence, we only included women with at least one pregnancy (and at least one preeclamptic pregnancy for the preeclampsia cases), in order to address the associations of preeclampsia, as well as genotype associations with hypertension. In order to avoid any potential immediate effects of the preeclamptic pregnancy on the association to postpartum hypertension, we selected individuals registered in MBRN that delivered with a diagnosis of preeclampsia at least one year prior to participation in HUNT2. Mean duration since the preeclamptic index pregnancy was 15.1 years (SD: 8.51, range: 1.0-31.0 years) at the time of inclusion in the HUNT2 study. Controls were matched for age to the index preeclampsia pregnancy.

We excluded individuals with missing information regarding whether or not they were taking any antihypertensive drugs at the time of enrollment in the HUNT2 study (not excluded in our previous paper studying risk of preeclampsia) [8] as well as missing information from MBRN of pregnancy duration at delivery. We also excluded women that at the time of index delivery in the MBRN had chronic hypertension, chronic renal disease, heart disease, or diabetes mellitus prior to pregnancy, and women with registered non-proteinuric gestational hypertension. The final study population consisted of 2945 women; 934 with a history of preeclampsia and 2011 women with a history of normotensive pregnancies.

The HUNT2 study was conducted according to the principles expressed in the Declaration of Helsinki. Attendance was voluntary, and each participant signed a written informed consent including information on genetic analyses. The study was approved by the Regional Committees for Medical and Health Research Ethics (REC Central), Norway.

### SNP analyses

DNA was extracted and rs4606 was analyzed with the TaqMan®-based single nucleotide polymorphism genotyping technology (assay ID: C__2498717_10) using 7900HT Fast RealTime PCR System (Applied Biosystems, Foster City, CA), as previously described [8].

### Statistical analyses

Data were analyzed using Predictive Analytics Soft Ware (version 18.0.1, SPSS Inc, Chicago, IL, USA). We applied the independent samples t-test to test for differences in continuous variables between groups and data are presented as mean and standard deviation (SD). Associations between categorical variables were tested by use of chi-square statistics (*X*^2^ test) or binary logistic regression. Proportions are given in percentages and risk estimates are given as odds ratios (OR) with 95% confidence intervals (CI).

We defined current hypertension (at HUNT2 Study) as systolic blood pressure ≥140 mmHg and/or diastolic blood pressure ≥ 90 mmHg and/or taking antihypertensive drugs, and used it as the outcome variable in logistic regression analysis. Additionally, we tested the outcome variables according to stage 2 hypertension, defined by the Seventh Report of the Joint National Committee on Prevention, Detection, Evaluation, and Treatment of High Blood Pressure (JNC7) [11]; SBP ≥ 160 mmHg and DBP ≥ 100 mmHg; separately, in combination and/or combined with those taking antihypertensive drugs. Classical CVD risk factors, as well as parity, a history of preeclampsia and the rs4606 SNP, were included as explanatory variables in the multivariate models.

Due to low numbers for some of the variables and categories, some of the categorical variables were re-categorized into fewer categories than the original coding. The three genotypes (CC, CG and GG) of rs4606, as well as merged into two categories (CC and CG + GG), were tested in separate models. In either case, the CC genotype was used as reference. Continuous variables in the main effects models were analyzed for linearity and categorized if indicated. Potential interaction terms were tested as the product between rs4606 genotypes and each of the other remaining explanatory variables in the main effects model. The final models were found sound when tested for Hosmer-Lemeshow goodness of fit (*P* > 0.05).

## Results

### Genotype distribution

As previously shown, the genotype distribution was significantly different between the preeclampsia group (with CC: 47.5% (n = 444); CG: 44.6% (n = 417); GG: 7.8% (n = 73)) compared to the control group (with CC: 53.5% (n = 1075); CG: 39.0% (n = 785); GG: 7.5% (n = 151)), *P* = 0.009 (the numbers differing slightly from our previous paper due to modified inclusion criteria, see Methods) [8].

### Clinical characteristics in women with a history of preeclampsia versus normotensive pregnancy

At inclusion in the HUNT2 study, known CVD risk factors were higher (BMI, lipids, blood pressure) or more prevalent (diabetes mellitus) in women with a history of preeclampsia compared to the control group (Table 1). Prevalence of current hypertension, including current use of antihypertensive drugs, was also higher in the group of women with previous preeclampsia compared to the control group (Table 1). The proportion of current smokers was higher in the control group compared to the previous preeclampsia group (Table 1). Age at inclusion in the HUNT2 study did not differ between the preeclampsia and the control group (Table 1). Women with a history of preeclampsia and current hypertension were younger than women with current hypertension and previous normotensive pregnancy (46.3 (SD: 9.65) years, versus 49.4 (SD: 10.0) years, *P* < 0.001).

**Table 1:** Clinical data at HUNT2 inclusion for women with a history of preeclampsia vs. control group

	**Preeclampsia**	**Control**	***P***
Age, mean (SD)	41.0 (9.81)	41.2 (10.8)	0.588
Parities, % (n)			
1	13.2 (123)	16.7 (336)	
≥ 2	86.8 (811)	83.3 (1675)	0.014
Pregnancy duration at delivery in weeks, mean (SD)	38.8 (2.76)	39.8 (2.29)	<0.001*
Delivery < 37 weeks gestation, % (n)	14.6 (136)	4.5 (91)	<0.001*
Delivery < 34 weeks gestation, % (n)	5.4 (50)	1.7 (34)	<0.001*
SBP, mean (SD)^†^	133.5 (19.7)	126.3 (17.6)	<0.001*
DBP, mean (SD)^†^	80.6 (11.9)	75.7 (11.0)	<0.001*
Hypertension, % (n)^‡^	37.7 (352)	21.7 (436)	<0.001*
SBP ≥ 140 mmHg, % (n)^§^	27.1 (228)	16.4 (316)	<0.001*
DBP ≥ 90 mmHg, % (n)^§^	18.1 (152)	8.2 (158)	<0.001*
SBP ≥ 160 mmHg, % (n)^§^	8.0 (67)	4.2 (81)	<0.001*
DBP ≥ 100 mmHg, % (n)^§^	5.0 (42)	2.2 (42)	<0.001*
Antihypertensive drugs: % (n)	Current: 9.9 (92)	Current: 4.3 (86)	
	Previous: 13.3 (124)	Previous: 1.60 (32)	
	Never: 77 (718)	Never: 94.1 (1893)	<0.001*
BMI, mean (SD)	27.9 (5.42)	25.8 (4.37)	<0.001*
Serum Cholesterol, mean (SD)	5.63 (1.12)	5.59 (1.24)	0.387
HDL, mean (SD)	1.45 (0.37)	1.49 (0.37)	0.022*
Total Cholesterol/HDL, mean (SD)	4.12 (1.36)	3.99 (1.40)	0.020*
Current smoker, % (n)	26.5 (234)	38.4% (741)	<0.001*
Diabetes, % (n)	3.1 (29)	1.0 (20)	<0.001*
CVD, % (n)	1.1 (10)	1.2 (25)	0.695

*Statistically significant *P* < 0.05. ^†^Regardless of taking antihypertensive drugs or not. ^‡^SBP ≥ 140 mmHg and/or DBP ≥ 90 mmHg and/or current use of antihypertensive drugs. ^§^Included in analysis only those women not pharmacologically treated for hypertension.

SBP: Systolic blood pressure. DBP: diastolic blood pressure. BMI: Body mass index. HDL: High density lipoprotein. CVD: Cardiovascular disease (= a current or past diagnosis of angina pectoris, myocardial infarction or cerebral stroke).

The clinical characteristics did not differ between the rs4606 genotype groups, neither in the total study population (preeclampsia + controls), nor within the preeclampsia and control group separately (all non-significant; data not shown).

### Risk of hypertension according to obstetric history and genotype

Table 2 shows the association between the rs4606 polymorphism and hypertension after pregnancy (in women that were without registered chronic disease prior to pregnancy). Results are given for the total study population, as well as for subgroups according to obstetric history. In addition to unadjusted results, minimally adjusted results with adjustment for age and age + BMI are given, as age and BMI are considered major predictors for hypertension. In unadjusted analysis, no significant association was found between any of the genotypes and hypertension at HUNT2 inclusion in the total study population (Table 2). However, a significant association was found between the GG genotype and hypertension after adjustment for age, and age + BMI (Table 2). A marginally significant association was also found between the GG genotype and hypertension in the group of women with a history of preeclampsia, whereas the association was weaker in the control group (Table 2). Based on the total study population, the association between the GG genotype and hypertension remained however significant if a history of preeclampsia was accounted for (OR (95% CI): 1.46 (1.02-2.09), *P* = 0.037), but was attenuated in a model adjusted for multiple other variables (main effects model with the following additional variables: total cholesterol/HDL cholesterol ratio, CVD, diabetes and hard physical activity; CG: OR (95% CI): 1.15 (0.91-1.46), *P* = 0.230; GG: OR (95% CI): 1.26 (0.82-1.94), *P* = 0.297). A history of preeclampsia remained an independent predictor for current hypertension also in the multivariate model, including adjustment for the rs4606 variable (OR (95% CI): 2.22 (1.76-2.80), *P* < 0.001).

**Table 2:** rs4606 and risk of hypertension (SBP ≥ 140 mmHg/DBP ≥ 90 mmHg and/or taking antihypertensive drugs) according to obstetric history

	**Unadjusted**			**Age-adjusted**			**Age and BMI-adjusted**		
**Obstetric history group**	**OR (95% CI)**^**†**^	***P***	**Number included in analysis (n)**	**OR (95% CI)**^**†**^	***P***	**Number included in analysis (n)**	**OR (95% CI)**^**†**^	***P***	**Number included in analysis (n)**
**Preeclampsia + control**	CG: 1.07 (0.90-1.27)	0.451	2945	CG: 1.10 (0.91-1.33)	0.321	2945	CG: 1.12 (0.92-1.36)	0.254	2937
	GG: 1.23 (0.90-1.67)	0.193		GG: 1.41 (1.01-1.97)	0.047*		GG: 1.49 (1.05-2.11)	0.027*	
**Control**	CG: 0.998 (0.80-1.25)	0.989	2012	CG: 1.07 (0.84-1.36)	0.604	2012	CG: 1.07 (0.83-1.38)	0.595	2007
	GG: 1.02 (0.68-1.54)	0.923		GG: 1.23 (0.78-1.92)	0.369		GG: 1.28 (0.80-2.04)	0.303	
**Preeclampsia**	CG: 1.07 (0.81-1.41)	0.653	933	CG: 0.98 (0.72-1.33)	0.897	933	CG: 1.03 (0.76-1.41)	0.838	930
	GG: 1.53 (0.93-2.52)	0.094		GG: 1.61 (0.93-2.80)	0.091		GG: 1.76 (1.00-3.11)	0.052	
**Early-onset preeclampsia**	CG: 3.30 (0.87-12.5)	0.079	50	CG: 4.50 (0.93-21.9)	0.062	50	CG: 7.90 (1.30-48.2)	0.025*	50
	CG/GG: 3.38 (0.90-12.6)^‡^	0.070		CG/GG: 4.68 (0.98-22.3)^‡^	0.053		CG/GG: 7.96 (1.33-47.8)^‡^	0.023*	
**Later onset preeclampsia**	CG: 1.01 (0.76-1.34)	0.946	883	CG: 0.913 (0.67-1.25)	0.570	883	CG: 0.95 (0.69-1.31)	0.761	880
	GG: 1.47 (0.88-2.43)	0.139		GG: 1.53 (0.87-2.68)	0.137		GG: 1.68 (0.94-2.98)	0.079	
**Recurrent preeclampsia**	CG: 1.98 (0.98-4.01)	0.058	158	CG: 1.73 (0.84-3.58)	0.139	158	CG: 1.66 (0.79-3.49)	0.183	158
	CG/GG: 2.04 (1.03-4.03)^ǂ^	0.040*		CG/GG: 1.78 (0.89-3.59)^ǂ^	0.106		CG/GG: 1.81 (0.88-3.71)^ǂ^	0.105	
**Non-recurrent preeclampsia**	CG: 0.95 (0.70-1.29)	0.747	775	CG: 0.871 (0.62-1.23)	0.428	775	CG: 0.94 (0.66-1.33)	0.710	772
	GG: 1.43 (0.82-2.49)	0.212		GG: 1.58 (0.84-2.98)	0.159		GG: 1.62 (0.85-3.12)	0.145	

*Statistically significant *P* < 0.05. SBP: Systolic blood pressure. DBP: Diastolic blood pressure. ^†^Reference genotype: CC. ^‡^Merging of CG + GG genotype (n = 28) due to low numbers in GG genotype group (n = 2).

^ǂ^Merging of CG + GG genotype (n = 91) due to low numbers in GG genotype group (n = 15). Non-recurrent preeclampsia: primiparas and multiparas with only one preeclamptic pregnancy.

When examining subgroups of preeclampsia, a significant association was found between the CG or GG genotype and hypertension in the early-onset (delivery prior to gestational week 34) preeclampsia group after adjustment for age and BMI (Table 2). In unadjusted analysis, a significant association was found between the CG or GG genotype and hypertension in the recurrent preeclampsia group (Table 2). No significant association was found between the genotypes and hypertension in the later onset (delivery from gestational week 34 and onwards) preeclampsia group or the non-recurrent preeclampsia group (Table 2). As some of the preeclampsia subgroups were very small (such as the early-onset and recurrent preeclampsia groups), limiting statistical power, further multivariate adjustment for an association between rs4606 and hypertension was not carried out for each of these sub-groups separately.

### Association between stage 2 hypertension, the rs4606 polymorphism and preeclampsia

In multivariate (but not in crude) analysis, we found a significant association between the rs4606 GG genotype and current stage 2 (severe) hypertension, defined as SBP ≥ 160 mmHg and/or current use of antihypertensive drugs. One-hundred-and-fifty-nine (16.8%) women with a history of preeclampsia were classified according to this definition of hypertension, compared to 170 (8.3%) women with a history of normotensive pregnancy (*P* < 0.001). Table 3 shows the association in the unadjusted model, a minimally adjusted model, a fully adjusted model (“multivariate model”) as well as a model containing only variables significant at 5% level (“main effects model”; variables excluded from the larger model were not found to be confounders for the association between the rs4606 variable and hypertension). A significant association for the same definition of hypertension was also found if the CG and GG genotypes of rs4606 were merged into one group (“main effects model”: OR (95% CI): 1.43 (1.02-2.00), *P* = 0.036). The association between rs4606 and stage 2 hypertension remained also significant if we added DBP ≥ 100 mmHg to the definition, but the polymorphism was not significantly associated with diastolic hypertension alone (data not shown). The association between the polymorphism and stage 2 systolic hypertension (as defined in Table 3) was also of same magnitude if analyses were carried out separately for women with a history of preeclampsia: OR (95% CI): CG: 1.36 (0.83-2.24), P = 0.224 and GG: 2.09 (0.90-4.87), P = 0.088 and controls: OR (95% CI): CG: 1.30 (0.79-2.13), *P* = 0.304 and GG: 1.82 (0.75-4.39), *P* = 0.184. Moreover, a history of preeclampsia remained a significant and independent predictor for current hypertension after multivariate adjustments, for all alternative criteria used in this study to define hypertension.

**Table 3:** Logistic regression model (outcome variable: systolic blood pressure ≥160 mmHg and/or taking antihypertensive drugs)

	***n***	**OR**	**95% CI**		***P***
			**Lower**	**Upper**	
**rs4606 genotype**^**‡ **^**(unadjusted)**	2944				
CG		1.03	0.81	1.31	0.834
GG		1.02	0.66	1.60	0.921
**Minimally adjusted model**^**#**^	2936				
rs4606 genotype^‡^					
CG		1.00	0.76	1.32	0.978
GG		1.16	0.69	1.93	0.582
**Multivariate model**^**†**^	2123				
rs4606 genotype^‡^					
CG		1.38	0.96	1.99	0.083
GG		1.88	1.02	3.47	0.044*
**Main effects model**^**§**^	2211				
rs4606 genotype^‡^					
CG		1.36	0.96	1.92	0.088
GG		1.93	1.05	3.53	0.033*
History of preeclampsia vs. control		3.27	2.32	4.60	<0.001*
Age		1.13	1.11	1.15	<0.001*
Hard Physical activity		0.58	0.39	0.88	0.010*
BMI ≥ 25		2.38	1.60	3.53	0.001*
Diabetes		5.14	2.31	11.4	<0.001*

*Statistically significant *P* < 0.05. ‡Reference: CC genotype. ^#^Age and BMI from HUNT2 and a history of preeclampsia were included in the minimally adjusted model in addition to the rs4606. †In addition to the rs4606, a history of preeclampsia and the following variables from HUNT2 were included in the multivariate model: age, CVD ever (= a current or past diagnosis of angina pectoris, myocardial infarction or cerebral stroke; Yes/No), Diabetes ever (Yes/No), Smoking cigarettes (Yes/No), Number of children (≥ 2 vs. 1), Hard physical activity (≥ 1 h/week vs. <1 h/week), BMI, Total Cholesterol/HDL-ratio. §Main effects model includes only variables significant at P < 0.05. BMI = Body mass index.

#### ***Interaction between rs4606 and physical activity***

Of the tested interactions, a significant interaction term was found between the combined CG and GG genotype group and hard physical activity with respect to current stage 2 hypertension (as defined in Table 3), with an OR of 3.09 (95% CI: 1.35-7.09), *P* = 0.008. The interaction term remained significant also if we adjusted for age at HUNT2 inclusion and obstetric history (preeclampsia versus normotensive pregnancy; data not shown). To investigate this further, we next stratified our analyses into those exercising 0- <1 h/week (named inactive women) and those exercising ≥1 h/week (named active women). We found that, in physically inactive women, the prevalence of hypertension did not differ significantly between those having the CC compared to those having the CG/GG genotype. In physically active women however, the prevalence of hypertension was significantly lower in CC versus CG/GG genotype carriers (Table 4). We found a similar trend if we did the same stratification, but defined hypertension as systolic blood pressure ≥140 mmHg and/or diastolic blood pressure ≥90 mmHg and/or current use of antihypertensive drugs (Table 5). In either case, the prevalence of hypertension was lower in physically active women as compared to physically inactive women, but this difference was statistically significant for CC genotype carriers only (Table 4 and Table 5).

**Table 4:** Prevalence of women with stage 2 hypertension according to rs4606 genotypes and physical activity/inactivity stratification

	**Systolic blood pressure ≥160 mmHg and/or taking antihypertensive drugs (% (n))**		
**Hard physical activity**	**CC**	**CG/GG**	***P***^**†**^
**0 - < 1 h/week**	10.0 (81)	11.2 (83)^§^	0.442
**≥ 1 h/week**	2.7 (9)	8.8 (29)^#^	0.001*
***P***^**‡**^	<0.001*	0.228	

*Statistically significant *P* < 0.05 (Pearson chi-square). ^†^CG/GG genotypes vs. CC genotype. ^‡^Physically active (≥ 1 h/week) vs. physically inactive (0 - < 1 h/week). ^§^Thereof GG genotype: n = 16. ^#^Thereof GG genotype: n = 3.

**Table 5:** Prevalence of women with hypertension according to rs4606 genotypes and physical activity/inactivity stratification

	**Systolic blood pressure ≥140 mmHg and/or diastolic blood pressure ≥90 mmHg and/or taking antihypertensive drugs (% (n))**		
**Hard physical activity**	**CC**	**CG/GG**	***P***^**†**^
**0 - < 1 h/week**	24.9 (202)	26.3 (195)^§^	0.545
**≥ 1 h/week**	17.5 (59)	22.4 (74)^#^	0.112
***P***^**‡**^	0.006*	0.171	

*Statistically significant *P* < 0.05 (Pearson chi-square). ^†^CG/GG genotypes vs. CC genotype. ^‡^Physically active (≥ 1 h/week) vs. physically inactive (0 - < 1 h/week). ^§^Thereof GG genotype: n = 30. ^#^Thereof GG genotype: n = 11.

## Discussion

In 2011, The American Heart Association added pregnancy complications such as preeclampsia to the list of risk factors for developing CVD [12]. A mechanistic explanation for the relationship between preeclampsia and later CVD is lacking [13]. We recently reported that women with a pregnancy complicated by preeclampsia differed in the genotype distribution of the rs4606 in *RGS2,* with a slightly higher proportion of women having either the CG or GG genotype, compared to women who never had a preeclamptic pregnancy [8]. This finding, along with an association between the G allele of rs4606, reduced *RGS2* expression and essential hypertension, as demonstrated by Semplicini et al. [7], led us to explore the possible association between this polymorphism and hypertension in women with a history of preeclampsia or uncomplicated pregnancy from our previously studied population. Our present findings support an association between the G allele of rs4606 and hypertension after pregnancy by showing that parous women have an increased risk of developing severe hypertension, in particular stage 2 systolic hypertension [11], if they have either the CG or GG genotype, compared to women with the CC genotype. Our data further showed that the association to current stage 2 systolic hypertension was strongest among individuals homozygous for the G allele, suggesting an allele dose–response relationship.

Merging of women with a history of preeclampsia and women without a history of preeclampsia is not without limitations, as these groups represent two highly selected populations, with preeclamptic women being at higher risk of later hypertension in general. Obstetric history was therefore accounted for in the regression model, and adjustment for a history of preeclampsia did not affect the significant relationship between genotype and hypertension. The magnitude of the association between the CG/GG genotypes and stage 2 hypertension was also similar in women with a history of preeclampsia and normotensive pregnancy if analyses were carried out separately for the two groups. The results did however not reach statistical significance in either group alone, which likely is due to limitations in statistical power resulting from the stratification of analyses. Our results also suggested a tendency towards a higher risk of milder hypertension defined as systolic blood pressure ≥140 mmHg and/or diastolic blood pressure ≥90 mmHg and/or current use of antihypertensive drugs in GG versus CC genotype carriers, with the strongest association in women with a history of preeclampsia. It is important to note that our study population was selected based on a previous diagnosis of preeclampsia or not, and not based on a retrospective case–control (hypertensives vs. normotensives) design. This could potentially have diluted an association between genotype and hypertension, especially in the control group. Our exclusion criteria, such as excluding women in the MBRN registered with pre-gestational chronic hypertension, renal disease, heart disease, any form of diabetes mellitus or non-proteinuric gestational hypertension, may also have diluted an association between genotype and hypertensive phenotype.

In our present study the association between the CG or GG genotype and stage 2 hypertension was detected only after multivariate adjustment and an interaction between rs4606 and physical activity was found. This is in line with remote hypertension after pregnancy being the result of multiple factors, with potential interaction effects. Previous studies have also suggested interactions between physical activity and genetics on blood pressure [14]. Moreover, it has been demonstrated that the blood pressure lowering effect of endurance training is greater in subjects with initial high blood pressures [14]. Results from our present study could therefore potentially suggest that in women with hypertension, and in particular severe, stage 2 hypertension, a more pronounced beneficial effect of physical activity on blood pressure reduction may be present in reference CC genotype carriers compared to CG or GG genotype carriers. An alternative interpretation is that despite being physically active, women carrying the CG or GG genotype will still be at a higher “baseline” (genetic) risk of developing hypertension compared to women carrying the CC genotype. In contrast, the elevated risk of hypertension associated with a sedentary lifestyle may dilute the difference in risk of hypertension that is seen between individuals with different genotypes, as those being “genetically protected” (i.e. CC genotype carriers) may significantly increase their risk of hypertension by being physically inactive, while physical inactivity may not add a similar risk in those already at risk (i.e. CG/GG genotype carriers). To our knowledge, no previous studies have investigated the potential interaction between this polymorphism (rs4606) and physical activity with respect to blood pressure reduction or the risk of hypertension, neither in women (whether parous or not), nor in men. Intervention studies are needed in order to elucidate this association. Nevertheless, all postpartum women should be encouraged to adhere to an active lifestyle, as physical activity in general is associated with a reduced risk of hypertension, in addition to being associated with a reduced risk of other lifestyle related conditions, such as obesity.

Several studies, including previous results from the HUNT study population, have shown that women with preeclampsia have more risk factors for CVD than women with uncomplicated pregnancies both pre-pregnancy and post-pregnancy [15,16], the latter also supported by our present study (Table 1). A history of preeclampsia remained however a strong predictor of hypertension after adjustment for the rs4606 genotype, age and other variables classically found to modulate CVD risk. This finding suggests that the preeclamptic event per se, or the presence of other risk factors not explored in the present study, may additionally increase the risk of hypertension later in life, and thereby the risk of CVD.

The RGS2 is a candidate for regulation of signaling through the Ang II type 1 receptor (AT1-receptor) [17,18]. Hercule et al. demonstrated that deletion of *RGS2* in mice resulted in an augmented response to Ang II and altered blood pressure regulation, indicating that signaling by G protein-coupled receptors is abnormally prolonged [6]. Several studies have shown that increased Ang II sensitivity and activating autoantibodies against the AT1-receptor (AT1-AA) are present during and after preeclampsia [19-23]. Future studies should investigate if the presence of AT1-AA influences the association of hypertension and rs4606.

There is epidemiological support for early-onset preeclampsia and recurrent preeclampsia being more strongly associated with CVD later in life than a diagnosis of preeclampsia in general [4]. Even in our relatively healthy and young parous population, we found in crude and minimally adjusted analyses respectively, that women with a history of recurrent-preeclampsia and early-onset preeclampsia were at a significantly increased risk of later developing hypertension (blood pressure ≥140/90 mmHg, and/or current use of antihypertensive drugs) if they carried the CG or GG genotype compared to the CC genotype. Unfortunately, the very low numbers of women in the recurrent preeclampsia and early-onset preeclampsia sub-groups limited further analyses on these groups, such as exploring other cut-offs for hypertension or performing comprehensive logistic regression analyses.

We have previously shown an association between the CG/GG genotype and the presence of uteroplacental acute atherosis in women with a preeclamptic pregnancy, with the strongest association in the early-onset preeclampsia group [8]. Acute atherosis is characterized by foam cell accumulation and lipid depositions in decidual segments of spiral arteries, resembling early stages of atherosclerotic disease [24]. Whether presence of acute atherosis in pregnancy could reflect an increased risk of atherosclerotic CVD in the woman later in life is currently unknown, but we believe a common pathogenesis is plausible [24]. Interestingly, Kamide et al. showed an association between intima media thickening of carotid artery and polymorphisms in *RGS2*, including 1891-1892del TC which is in linkage disequilibrium with the functional variant rs4606 [25]. In the current study, based on the HUNT2 population, a diagnosis of uteroplacental acute atherosis and risk of future hypertension or CVD could not be explored, as this is not a routine clinical examination after delivery. We were also not able to explore an association between rs4606 and clinical CVD other than for hypertension, as our present study included very few clinical cardiovascular endpoints, likely due to a relatively young age group at HUNT2 inclusion, with a mean age of 41 years. Women with clinical CVD may also self selectively have been underrepresented in the HUNT2 study. Although a genetic predisposition for hypertension may be more evident for the younger age group, we cannot rule out the possibility of stronger associations in general, or according to pregnancy history, between our genotypes studied and hypertension in an older and less healthy (at parity) population.

Some caution is needed when interpreting our findings. Information on whether or not the study subjects were taking antihypertensive drugs was based on self-report. The definition of hypertension in those reporting no use of antihypertensive drugs was based on readings at only one time point (at enrollment in the HUNT2 study), using the average of the two last of three blood pressure readings. In a clinical setting, a diagnosis of hypertension, and the potential start of pharmacological treatment, is usually based on repeated measures of elevated blood pressure. Physical activity was also self-reported, based on a short questionnaire. A validation study of the physical activity questionnaire in the HUNT2 study has previously been carried out [26], and the “hard physical activity” question was found to be a useful measure of vigorous physical activity. In contrast, based on results from this report, we did not include the answer option “light” physical activity in our analyses, as this question was found to have poor reproducibility and to have poor correlation with most comparison measures [26]. Total physical activity level was therefore not accounted for in the present study. Additionally, for our study, several women (approx. 700) were lost in the regression analysis due to missing data for the hard physical activity question. Another limitation of our study may be the sampling of blood in the non-fasting state in the HUNT2 study. We decided to include the ratio of total cholesterol to HDL in our analyses, as it has been shown that serum concentrations of these lipid compounds are little influenced by recent food intake [27]. Inclusion (as in the multivariate model) or exclusion (as in the main effects model) of this variable did however not influence the association shown between the rs4606 and stage 2 hypertension. Despite these limitations, a major strength of our study is a well-described population-based cohort (HUNT2), with selection of a healthy parous subgroup (except for preeclampsia in the case group) and a large total sample size, enabling the adjustment for several important postpartum follow-up variables in the HUNT study, such as age, BMI, smoking, parities and postpartum diabetes.

## Conclusion

In our present study we have shown that the rs4606 CG/GG genotypes of *RGS2*, in particular the GG genotype, are associated with hypertension in women after pregnancy, even after excluding women who had a pregnancy record of chronic hypertension, cardiac or renal disease or pregestational diabetes mellitus. Our study in a relatively healthy population therefore strengthens a previously reported association between the G allele of this polymorphism and essential hypertension, and for the first time demonstrates an association between this polymorphism and hypertension outside of pregnancy in a Norwegian population. As the CG or GG genotypes are more prevalent in women with a history of preeclampsia compared to women with a history of normotensive pregnancy, these genotypes may represent one piece of a puzzle for the epidemiologically reported higher prevalence of hypertension later life in women with preeclampsia. Dysregulated angiotensin signaling could be a potential mechanistic link that merits further investigation. Thus, this polymorphism in the RGS2 gene may be a common predisposing factor for the development of hypertension in pregnancy (preeclampsia) and hypertension outside of pregnancy. The present study also support a multifactorial relationship between preeclampsia and an increased risk of hypertension later in life, as a history of preeclampsia remained a strong and independent risk factor for hypertension after accounting for this polymorphism and classical risk factors for CVD, such as BMI, age and diabetes. The relative contributions from genetic factors, environmental factors and pregnancy factors on the long term risk of hypertension after preeclampsia needs further examination, and may also differ between populations as well as within populations.

## Competing interests

The authors’ declare that they have no competing interests.

## Authors’ contributions

ASK has conceived and designed the research, analyzed and interpreted the data, performed statistical analyses, drafted and revised the manuscript critically for important intellectual content*.* ØM has conceived and designed the research, analyzed and interpreted the data and revised the manuscript critically for important intellectual content. OLH has analyzed and interpreted the data, performed statistical analyses and revised the manuscript critically for important intellectual content. HL has analyzed and interpreted the data and revised the manuscript critically for important intellectual content*.* RD has analyzed and interpreted the data and revised the manuscript critically for important intellectual content. ACS has conceived and designed the research, analyzed and interpreted the data, as well as supervised the research, and has revised the manuscript critically for important intellectual content. All authors read and approved the final manuscript.

## Pre-publication history

The pre-publication history for this paper can be accessed here:

http://www.biomedcentral.com/1471-2350/15/28/prepub
